# Reviewer Acknowledgement 2013

**DOI:** 10.1186/1546-0096-12-7

**Published:** 2014-02-03

**Authors:** Alberto Martini, Charles Spencer

**Affiliations:** 1 University of Genoa, Genoa, Italy; 2 Ohio State University, Columbus, USA

## Abstract

**Contributing reviewers:**

The Editors of *Pediatric Rheumatology* would like to thank all our reviewers who have contributed to the journal in Volume 11 (2013).

## 

Navid Adib

Australia

Amita Aggarwal

India

Roger Allen

Australia

Mariane Amano

Brazil

Jordi Anton

Spain

Wineke Armbrust

The Netherlands

Tadej Avcin

Slovenia

Eileen Baildam

United Kingdom

Jennifer Barton

United States of America

Radovan Bogdanovic

Serbia

Mortaza Bonyadi

Iran

Victoria Borba

Brazil

Riva Brik

Israel

Paul Brogan

United Kingdom

Diane Brown

United States of America

Juergen Brunner

Austria

Ruben Burgos-Vargas

Mexico

David Cabral

Canada

Anne Marie Cahill

United States of America

Laurent Camous

France

Sabrina Cavallo

Canada

Omkar Chaudhary

India

Nadine Choueiter

United States of America

Rolando Cimaz

Italy

Tamás Constantin

Hungary

Randy Cron

United States of America

Megan Curran

United States of America

John Dagle

United States of America

Pavla Dolezalova

Czech Republic

Jean-Charles Doucet

Canada

Ciaran Duffy

Canada

Justine Ellis

Australia

Fernanda Falcini

Italy

Francis Fatoye

United Kingdom

Polly Ferguson

United States of America

Ivan Foeldvari

Germany

Helen Foster

United Kingdom

David Fox

United States of America

Marco Gattorno

Italy

Abraham Gedalia

United States of America

Hermann Girschick

Germany

Rodney Grahame

United Kingdom

Thomas Griffin

United States of America

Aysen Gunel-Ozcan

Turkey

Kathleen Haines

United States of America

Keri Hainsworth

United States of America

Liora Harel

Israel

Troels Herlin

Denmark

Anne Hinks

United Kingdom

Michaël Hofer

Switzerland

Gerd Horneff

Germany

Toni Hospach

Germany

Adam Huber

Canada

Haku Iizuka

Japan

Roman Jurencak

Canada

Pentti Kallio

Finland

Azza Mahmoud Kamel

Egypt

Christian Kellenberger

Switzerland

Susan Kim

United States of America

Yukiko Kimura

United States of America

Marisa Klein-Gitelman

United States of America

Susan Klepper

United States of America

Isabelle Kone-Paut

France

Cengiz Korkmaz

Turkey

Kaisu Kotaniemi

Finland

Helen Lachmann

United Kingdom

Pekka Lahdenne

Finland

Bianca Lang

Canada

Thomas J A Lehman

United States of America

Deborah Levy

Canada

Carol Lindsley

United States of America

Daniel Lovell

United States of America

Silvia Magni-Manzoni

Italy

John Mahan

United States of America

Isabelle Marc

Canada

Alberto Martini

Italy

Liza Mccann

United Kingdom

Michael Miller

United States of America

Diana Milojevic

United States of America

Kirsten Minden

Germany

Lakshmi Moorthy

United States of America

Masaaki Mori

Japan

Mary Jo Mulligan-Kehoe

United States of America

Bahram Namjou

United States of America

Rob Nickeson

United States of America

Ellen Nordal

Norway

Sheila Oliveira

Brazil

Michael Ombrello

United States of America

Kathleen O’neil

United States of America

Karen Onel

United States of America

Ozan Ozkaya

Turkey

Anjali Patwardhan

United States of America

Thomas Klit Pedersen

Denmark

Evangeline Pillebout

France

Oscar Porras

Costa Rica

Sampath Prahalad

United States of America

Berent Prakken

Netherlands

Pierre Quartier

France

Egla Rabinovich

United States of America

Athimalaipet Ramanan

United Kingdom

Angelo Ravelli

Italy

Jutta Richter

Germany

Lisa Rider

United States of America

Sarah Ringold

United States of America

Johannes Roth

Germany

Ricardo Russo

Argentina

Helga Sanner

Norway

Rotraud Saurenmann

Switzerland

Lone Schejbel

Denmark

Christiaan Scott

South Africa

Alper Soylu

Turkey

Anne Helene Spannow

Denmark

Joost Swart

Netherlands

Yilmaz Tabel

Turkey

Kei Takahashi

Japan

Melissa Tesher

United States of America

Andrea Tesija Kuna

Croatia

Susan Thompson

United States of America

Kjell Tullus

United Kingdom

Marinka Twilt

Canada

Catrin Tzaribachev

Germany

Yosef Uziel

Israel

Janjaaap van der Net

The Netherlands

James W. Varni

United States of America

Jelena Vojinovic

Serbia

Linda Wagner-Weiner

United States of America

Carol Wallace

United States of America

Peter Weiser

United States of America

Patricia Woo

United Kingdom

Nico Wulffraat

United Kingdom

Shumpei Yokota

Japan

Xuan Zhang

China

Gaston Zilleruelo

United States of America

Francesco Zulian

Italy

